# Text message reminders to improve questionnaire response rates in RCTs: findings from three randomised sub-studies

**DOI:** 10.1186/1745-6215-16-S2-P103

**Published:** 2015-11-16

**Authors:** Ada Keding, Sally Brabyn, Hugh MacPherson, Stewart Richmond, David Torgerson

**Affiliations:** 1 University of York, York, UK; 2 Sydera Research Associates, Market Weighton, UK

## Background

Valid treatment effect estimates in the analysis of RCTs using patient reported outcomes depend on adequate response rates. Losses to follow-up are often high, and inexpensive ways to improve retention are much sought.

## Aim

To assess the effectiveness of reminders sent by SMS text messages before or after questionnaire distribution on patient response rate and time to response in a mental health trial population.

## Methods

Three randomised sub-studies were embedded in the UK ACUDep trial at three follow-up points. 523 patients of 755 in the main trial consented to being contacted by text message and were randomised to a pre-questionnaire reminder or no reminder at 3 months, a pre-reminder or post-reminder at 6 months and a post-reminder or no reminder at 9 months. Chi square tests and time-to event analyses were used to assess attrition between groups.

## Results

Return rates for pre-reminder SMS were not significantly different at 3 months compared to no reminder (82.9% vs 84.7%, p=.580), but showed significantly lower response rates at 6 months compared to post-reminders (75.2% vs 83.2%, p=.025). Return rates following post-reminders did not significantly differ at 9 months from no reminders (77.1% vs 78.5%, p=.691). Median times to response ranged from 18 to 25 days, with only returns at 6 months being significantly superior for patients receiving post-reminders (log-rank test p=.044).

## Conclusions

Overall, SMS text reminders did not appear to substantially improve patient response rates, although the pattern of effects for reminders sent before or after questionnaire distribution was inconclusive.

